# The Efficiency of Sclerotherapy in the Treatment of Vascular Malformations: A Retrospective Study of 63 Patients

**DOI:** 10.1155/2016/2809152

**Published:** 2016-12-15

**Authors:** Esko Veräjänkorva, Riitta Rautio, Salvatore Giordano, Ilkka Koskivuo, Otto Savolainen

**Affiliations:** ^1^Department of Surgery, Division of Plastic Surgery, Turku University Hospital, Turku, Finland; ^2^Department of Radiology, Turku University Hospital, Turku, Finland

## Abstract

*Background and Aims. Vascular* malformations are a vast group of congenital malformations that are present at birth. These malformations can cause pain, pressure, and cosmetic annoyance as well as downturn growth and development in a child in the case of* high flow*. Sclerotherapy has become an important tool in the treatment of vascular malformations. However, little is known about the success rate of sclerotherapy.* Material and Methods*. In this study, the efficiency of sclerotherapy in the treatment of vascular anomalies was investigated retrospectively in 63 patients treated in Turku University Hospital between 2003 and 2013.* Results*. Out of the 63 patients investigated, 83% (53) had venous malformations (VMs) and 9% (5) were defined as having arteriovenous malformations (AVMs). Patients with a VM were operated on, in 14% (8) out of all VM cases. Hence 86% (45) of patients with a VM received adequate help to their symptoms solely from sclerotherapy. The duration of treatment for the 14% of the VM patients that needed a surgical procedure was prolonged by 7–9 months, that is, by 41%.* Conclusions*. Sclerotherapy is an effective method in the treatment of VMs with a satisfactory clinical response in patients symptoms in 84% of cases.

## 1. Introduction

It is estimated that the prevalence of vascular malformations (VMs) in the population is around 4,5% [1]. VMs are congenital vascular malformations (CVMs) that are classified according to anatomical, pathological, and embryological criteria [2]. The most used classification system is the Hamburg classification (also known as the ISSVA classification) from 1988 and it has become the standard system in classification of congenital vascular malformations. This classification has since been updated in Colorado in 1992 and again in Rome in 1996 [3]. This system separates the malformations into arterial malformations (AMs), venous malformations (VMs), arteriovenous malformations (AVMs), lymphatic malformations (LMs), and capillary malformations (CMs) and combined vascular defects. These malformations are known to manifest in all parts of the human body. In addition, these malformations are present at birth; that is, they are congenital, but they usually induce clinical symptoms and findings after childhood, in early adulthood, or in later state of life by the influence of various factors such as trauma, infection, or hormones [4].

Vascular malformations can cause a variety of symptoms depending on their anatomical locations as well as on the flow characteristics of the malformation. It is important to distinguish the different vascular anomalies from each other since the treatment of each type of anomaly differs from the other [5]. A vascular malformation that has an arterial blood pressure (a so-called* high-flow* malformation, AM and AVM) is usually characterized by pain and a sense of pressure. In pediatric patients a high-flow malformation such as AMs and AVMs can cause a downturn of growth and development since the malformation steals blood from the circulation [6].* Low-flow* malformations such as venous malformations (VMs) and lymphatic malformations (LMs) cause also problems such as dripping of lymphatic fluid or blood through skin and pain, inflict cosmetic annoyance, and exposes the patient to infection [4].

Diagnosis of a vascular malformation is primarily clinical, but ultrasound and especially magnetic resonance imaging (MRI) has an important role [1] in the diagnosis and characterization of the lesion. The treatment of an individual patient is evaluated by a multidisciplinary team that should be centralized in hospitals that have adequate patient population. Treatment options can include minimal therapies such as elevation, compression garments, and aspirin whereas medical management of LMs can require antibiotics and steroids [7]. However, the assessment whether to use surgical or interventional radiologic techniques requires individual evaluation with a multidisciplinary approach and is determined by several factors such as the anatomical site of the lesion, patient expectations, and the facilities at hand in a given hospital. In this judgement the Hamburg classification provides a valuable instrument [8]. Absolute indications for treatment of the CVMs include hemorrhage and hemodynamic problems such as high-output cardiac failure or secondary ischemic complications caused by high-flow AV shunting [9].

Sclerotherapy has become an important tool in the treatment of vascular malformations. However, there has not been presented any evidence that some single sclerosing agent is preponderant if efficiency compared to other products in clinical trials; thus the radiologist personal preference does play a role in the selection of the sclerosant agent [10]. Sclerotherapy is conducted by a radiologist in ultrasound guidance by an injection of a sclerosant substance intravenously, such as polidocanol (Aethoxysklerol®). Polidocanol induces endothelial damage, inflammation, and eventually thrombosis of the vessel. This measure thus causes either a total or partial atrophy of the malformation. The effect of sclerotherapy can be evaluated two months after the injection. Sclerotherapy of VM is in general well tolerated. It can induce some local pain and swelling. In rare cases it can cause necrosis of the skin and nerve injury [11]. LMs are handled by an injection of avirulent* Streptococcus Pyogenes* bacteria (*OK-432*, Picibanil®). The injection of bacteria induces a strong inflammation inside the lymphatic vessel thus inducing atrophy of the vessel. CMs are often treated by light or laser treatment [12].

High-flow malformations such as AMs and AVMs on the other hand are treated with surgical excision. However, prior to surgical excision the high-flow malformation is embolized by a radiologist. In this procedure a sclerosant is injected inside the vessel, for example, gelatin, ethanol, or ethylene vinyl alcohol (Onyx®) [13]. In vast majority this results into tissue necrosis and therefore a surgical resection is mandatory after embolization.

VMs are often complex in structure and penetrate through many tissue structures. Thus radical surgical removal of a vascular malformation would often result in too excessive procedure and tissue morbidity. In addition, the vascular malformation is likely to relapse in case of intralesional excision. Therefore, in the case of VMs, the method of treatment is sclerotherapy. Only if repeated sclerotherapies are unsuccessful, a surgical excision can be performed [14].

Despite the vast research and the number of publications made in the field of sclerotherapies, there is not any study made on the effectiveness of sclerotherapies as a monotherapy in the treatment of vascular malformations. In this study, the success rate of sclerotherapy on the treatment of vascular malformations was investigated retrospectively in patients treated in Turku University Hospital between 2003 and 2013.

## 2. Materials and Methods

The material for this study was gathered from the patients treated with sclerotherapy for vascular malformation in the Turku University Hospital between 2003 and 2013. The material covers for 63 consecutive patients. The journals of each patient were examined for the following factors: age, medical specialty in charge of treatment, sporadic or familiar malformation, single or multiple and anatomic lesions, any prior treatment, type of radiological imagining, nature of the malformation (venous, lymphatic, venolymphatic, capillary or arteriovenous), smoking, number of sclerotherapies, nature of the sclerosant that was used (polidocanol, OK-432, ethanol, and glue), complications, and duration (follow-up) of treatment.

These factors were recorded in an Excel program and analyzed using the SPSS statistics program. The aim of the analysis was to find predisposing factors that will predict poor outcome in sclerotherapy. Permission for the study was granted by the Head of Department of Surgical Operations and Oncology in the University Hospital of Turku (permission number T182/2013).

## 3. Results

The 63 patients were divided into two groups: patients that eventually underwent a surgical procedure* versus* patients that did not. Patients were decided to be operated on if the result of the sclerotherapy was regarded as poor. These two patient groups were compared regarding the factors presented above (see Section 2) for statically significant differences.

### 3.1. Demographics

In the Turku University Hospital there were 63 patients treated with sclerotherapy for vascular malformation between 2003 and 2013.

In this study, neither gender nor age was associated to be a predisposing factor for a poor result in sclerotherapy (Table 1).

### 3.2. Surgical Specialty

The treatment of the 63 patients was divided between branches of surgical specialties. Majority of the patients, 37 (59%), were treated by plastic surgeons, nine (14%) were treated by pediatric surgeons, six (10%) were treated by hand surgeons, two (3%) were treated by neurosurgeons, and one (2%) was treated by ENT surgeon. The surgical specialty that managed the treatment of the patient was not associated to be a predisposing factor for a poor result in sclerotherapy.

### 3.3. Family History

There was evidence of family history only in the case of one patient; thus this patient was regarded as having familiar venous malformation. This particular patient was treated altogether by 12 times of sclerotherapies and eventually no surgery was performed. Hence, the nature of the malformation (sporadic* versus* familiar) was not associated to be a predisposing factor for a poor result in sclerotherapy.

### 3.4. Smoking

The history of smoking was poorly documented in the cases of nearly all patients. Therefore smoking could not be proven to be a predisposing factor for a poor result in sclerotherapy.

### 3.5. Single* versus* Multiple Lesions

Single vascular malformations covered 80% (50 patients) out of all patients; that is, in 20% of patients (12) the malformations were multiple. From the patients that were operated on (12), only one had multiple (*low*-flow) lesions. Thus whether the malformation was single or multiple, there was no association for a poor result in sclerotherapy.

### 3.6. Anatomical Location

The anatomical distribution of the malformations is illustrated in Table 2.

In this study, the anatomical location of the malformation was not associated to be a predisposing factor for a poor result in sclerotherapy.

### 3.7. History of Previous Treatment

In this study, patient was regarded to have previous medical treatment for vascular malformation, if at least one treatment event could be indicated in their history prior to the latest course of treatment. Out of the patients investigated, 30% had a history of previous treatment for their vascular malformation. Out of the patients that were eventually operated on, 50% had a history of previous treatment for their vascular malformation. In all cases the prior treatments were previous attempts of sclerotherapy.

### 3.8. Identification and Classification of Malformations

Identification of the malformation was done by MRI imaging in 87% (55 patients) of cases whereas ultrasound was used in 13% (8) of cases. Venous malformations covered 83% (52 patients) of all malformations and respectively 9% (5) were classified as arteriovenous malformations. No capillary, lymphatic, or venolymphatic malformations were identified in this study material. However, in 8% (5 patients) of the cases the precise nature of the malformations was left uncertain.

### 3.9. Sclerotherapy Methodology

Sclerotherapy was conducted 2,39 (median 2, range 1–12) times to patients that were not needed to operate on, but patients who were eventually operated on received sclerotherapy 3,25 (median 3, range 1–12) times. The sclerosant agent used before 2008 was ethanol, after which mainly polidocanol was used.

However, 25% of the patients that eventually were operated on reported also prolonged pain in the operation area. See Table 3 for all complications reported. Majority of patients stayed at the hospital for one night and were discharged on the following morning after sclerotherapy. No general anesthesia was needed to conduct the sclerotherapies in any of the patients.

### 3.10. Success Rate in Sclerotherapy

From the 63 patients studied in this study, 20% (12) were eventually operated on. This includes also all the patients with a high-flow malformation (5). Patients with a low-flow malformation were operated on, in 14% (8 patients) of cases. Thus 86% of patients with a low-flow malformation received sufficient alleviation to their symptoms from sclerotherapy.

### 3.11. Duration of Treatment

Duration of treatments (i.e., follow-up) in all patents was 21 months on average (median 17 months, range 2–89). Treatment duration in patients that did not need surgery was 20 months (median 14, range 2–89). Treatment duration in patients that eventually underwent surgery was 29 months (median 24, range 3–72). The difference of treatment duration between operated on and non-operated on patients is therefore 9 months and it infolds a statistically significant difference between these two groups (*p* = 0,035).

## 4. Discussion

In this study, the sclerotherapy was found to be an efficient method to relieve the subjective symptoms of a patient with VMs. From the 63 patients studied in this study, 20% (12) were eventually operated on. However this includes also all the patients that had AM or AVM (5). Patients with a VM were operated on, in 14% (8 patients) of cases with a venous malformation. This means that 86% of patients with a VM received adequate help to their symptoms solely from sclerotherapy. This is in level with earlier reports of the efficiency of sclerotherapy in the treatment of VMs [14, 15]. However, all AMs and AVMs did receive preoperative sclerotherapy before operation which is today considered the proper treatment protocol of such malformations [16].

The indication for sclerotherapy in the cases of VMs is mainly the symptoms that the patient has (such as pain or a sensation of a lump or a true deformity); that is, it is rather subjective. However, in the case of AVMs the indication is medically more objective since those malformations are prone to create risks and problems (such as stealing blood from the circulation) but also have more severe symptoms. Consistently the vascular therapy was considered successful if the patient subjectively experienced that the previous symptoms had been discharged through the sclerotherapy.

In this study, there was only one actual complication reported: a necrosis of finger that had to been amputated. This complication occurred as a result of the anatomical fact that there is quite limited vascular supply in fingers. Hence compromising the blood circulation by any means, such as with sclerotherapy, always infolds a risk of tissue necrosis. This is also irrelevant to the type of sclerosant used. This patient was not included in the group of operated on patients. However, there were some adverse events reported in the cases of 10 patients (16%). These included pain (8 patients) and fever (one patient) as well as haematoma in one patient. This result amplifies the consumption of sclerotherapy as a safe treatment option for vascular malformations.

In this study the efficiencies of each individual sclerosant agent were not compared with each other. In a vast review study [17] the efficiencies of different sclerosants used in 1552 patients were compared (from 36 articles). However, despite the strength of that study, the review failed to identify an optimal sclerosant agent. Thus it is not consistent to expect that this notably narrower study (63 patients) could manage to indicate differences between the sclerosants in terms of either efficiency or complication frequency. With similar reasoning, sclerotherapies of the AVMs were not profoundly subanalyzed since there were only five cases of such patients infolded in this study.

Despite the fact that sclerotherapy is not a treatment that radically abolishes a VM, sclerotherapy still manages to reduce the size of a VM, hence reducing symptoms. In addition, sclerotherapy is less invasive procedure than surgical operation, thus causing less tissue morbidity. Sclerotherapy has been estimated to be successful in 75–90% of cases. However, a single sclerotherapy is seldom sufficient for an adequate treatment response. Therefore, sclerotherapy needs often to be applied several times before satisfactory response has been obtained [14]. In this study, sclerotherapy was applied approximately 2,39 (median 2, range 1–12) times to those patients that did not eventually go through surgical procedure; that is, sclerotherapy was considered sufficient. This covers 86% of the patients with a VM. The patients that did not receive adequate treatment response solely with sclerotherapy and underwent surgery received sclerotherapy 3,25 (median 3, range 1–12) times. In this study, no statistical significance of patient age, family history, anatomical localization, the number of malformations, or the number of sclerotherapies could be found to explain or correlate with poor treatment response of sclerotherapy.

In this study the radiological size of the lesion was not marked up. However, this information was not provided in all three-dimensional values and thus the true volume of the malformations could not been calculated. In addition, the radiologist in hand was not always the same as the data had been collected through a period of ten years; thus there was reckoned change which also induced imprecision between the reported sizes of the lesions. In future prospective studies with more standardized and systematic volume calculation protocol it may be possible to evaluate whether the size of the lesions predicts bad prognosis for sclerotherapy. However, such a research question will demand a large volume of patients.

The median of treatment durations (i.e., follow-up time) in patients that were successfully treated solely with sclerotherapy was 14 months. In patients that were eventually operated on the median time was 24 months. There is a statistically significant difference in these durations (*p* < 0,035).

In conclusion it can be stated that sclerotherapy is a well tolerated and sufficient method in the treatment of VMs with a success rate of over 86%. In patients that will need complementary surgery, the duration of treatment lengthens by 7–9 moths, that is, by 41%.

## Figures and Tables

**Table 1:** 

Gender	
Male	23 (37%)
Female	40 (63%)
Operated on patients (all)	12 (19% out of *high- and low-flow* malformations combined)
Operated on males	4 (17% out of women)
Operated on females	8 (20% out of men)
Operated on *low-flow *malformations	8 (14% out of all *low-flow *malformations)
Average age of all patients	36
Median age of all patients	33
Range of age of all patients	3–88
Average age of operated on patients	30
Median age of operated on patients	30
Range of age of operated on patients	6–67

**Table 2:** Anatomical location.

Lower extremity	36 patients (57%)
Upper extremity	15 (24%)
Head and neck	8 (13%)
Torso	3 (5%)
Multiple locations	1 (2%)

**Table 3:** Complications: 10 patients (16%).

Pain	8 (13%)
Fever	1 (2%)
Haematoma	1 (2%)
Finger necrosis	1 (2%)

## References

[1] Greene A. K. (2011). Vascular anomalies: current overview of the field. *Clinics in Plastic Surgery*.

[2] Lee B. B., Laredo J., Lee T. S., Huh S., Neville R. (2007). Terminology and classification of congenital vascular malformations. *Phlebology*.

[3] Legiehn G. M., Heran M. K. S. (2006). Classification, diagnosis, and interventional radiologic management of vascular malformations. *Orthopedic Clinics of North America*.

[4] Azizkhan R. G. (2013). Complex vascular anomalies. *Pediatric Surgery International*.

[5] Garzon M. C., Huang J. T., Enjolras O., Frieden I. J. (2007). Vascular malformations. Part I. *Journal of the American Academy of Dermatology*.

[6] Greene A. K., Orbach D. B. (2011). Management of arteriovenous malformations. *Clinics in Plastic Surgery*.

[7] Buckmiller L. M. (2004). Update on hemangiomas and vascular malformations. *Current Opinion in Otolaryngology and Head and Neck Surgery*.

[8] Lee B. B. (2005). New approaches to the treatment of congenital vascular malformations (CVMs)—a single centre experience. *European Journal of Vascular and Endovascular Surgery*.

[9] Lee B.-B., Bergan J. J. (2002). Advanced management of congenital vascular malformations: a multidisciplinary approach. *Cardiovascular Surgery*.

[10] de Lorimier A. A. (1995). Sclerotherapy for venous malformations. *Journal of Pediatric Surgery*.

[11] Dubois J., Soulez G., Oliva V. L., Berthiaume M.-J., Lapierre C., Therasse E. (2001). Soft-tissue venous malformations in adult patients: imaging and therapeutic issues. *Radiographics*.

[12] Ernemann U., Kramer U., Miller S. (2010). Current concepts in the classification, diagnosis and treatment of vascular anomalies. *European Journal of Radiology*.

[13] Pekkola J., Lappalainen K., Vuola P., Klockars T., Salminen P., Pitkäranta A. (2013). Head and neck arteriovenous malformations: results of ethanol sclerotherapy. *American Journal of Neuroradiology*.

[14] Burrows P. E., Mason K. P. (2004). Percutaneous treatment of low flow vascular malformations. *Journal of Vascular and Interventional Radiology*.

[15] Lee B. B., Do Y. S., Byun H. S., Choo I. W., Kim D. I., Huh S. H. (2003). Advanced management of venous malformation with ethanol sclerotherapy: mid-term results. *Journal of Vascular Surgery*.

[16] Greene A. K., Alomari A. I. (2011). Management of venous malformations. *Clinics in Plastic Surgery*.

[17] Horbach S. E., Lokhorst M. M., Saeed P., de Goüyon Matignon de Pontouraude C. M., Rothová A., van der Horst C. M. (2016). Sclerotherapy for low-flow vascular malformations of the head and neck: a systematic review of sclerosing agents. *Journal of Plastic, Reconstructive & Aesthetic Surgery*.

